# Establishment of human-relevant *in vitro* models using animal-free serum replacement and recombinant antibodies

**DOI:** 10.3389/ftox.2026.1741716

**Published:** 2026-03-20

**Authors:** Zahra Miri, Johanna Laakkonen, Emilia Toivonen, Niina Väljä, Susanna Miettinen, Hanna Vuorenpää

**Affiliations:** 1 Adult Stem Cell Group, Faculty of Medicine and Health Technology, Tampere University, Tampere, Finland; 2 Tays Research Services, Tampere University Hospital, Wellbeing Services County of Pirkanmaa, Tampere, Finland

**Keywords:** animal-free blocking, bovine serum albumin, *in vitro* models, non-specific binding, recombinant antibody, serum replacement

## Abstract

The use of animal-derived reagents in biomedical research poses challenges for reproducibility due to batch-to-batch variability and inter-species differences, along with ethical concerns related to their origin. In pursuing a human-relevant *in vitro* model, an animal-free and defined cell culture process is preferred to improve relevance and reproducibility. We investigated the use of serum replacement (SR) consisting of human hepatocyte-derived proteins in cell culture and recombinant antibodies with a plant-derived blocking solution (animal-free blocker, AFB) in immunocytochemical staining of cells. Human serum (HS) instead of animal-derived serum was used in this study for comparison with SR. We showed that bone marrow stem/stromal cells (BMSCs) maintain their proliferation capacity and cell-specific morphology in SR-supplemented medium, whereas human umbilical vein endothelial cells (HUVECs) show compromised growth under similar conditions. In a more complex co-culture, BMSCs + HUVECs formed a stable vascular network in SR-supplemented medium. In immunocytochemical staining, we compared the performance of recombinant antibodies with animal-derived antibodies and an AFB solution with a bovine serum albumin (BSA)-based blocking solution. Adipose stem/stromal cells (ASCs) showed their typical spindle-shaped morphology when stained with recombinant antibodies against alpha-smooth muscle actin (αSMA) in both AFB and BSA-based blocking solutions. We detected partial non-specific binding of recombinant antibodies and animal-derived antibodies against β-tubulin III in ASC. In contrast, we did not observe non-specific binding on these neuronal antibodies in HUVECs in any tested condition. While protocol optimization depends on the cell type used, our findings indicate that animal-derived materials can reliably be replaced.

## 1 Introduction

Biomedical research aims to understand the biology and basis of human diseases to improve the diagnosis and treatment of various illnesses. Since most of the preclinical research cannot be conducted directly on humans, researchers have developed varying model organisms to mimic and reproduce human biology and diseases. Animal models, particularly transgenic mice, are often employed for this purpose (Benam et al., 2015). Although many animal models reproduce disease symptoms and phenotypes that appear similar to humans, studies have shown that the molecular mechanisms underlying these conditions can differ significantly (Seok et al., 2013; Cardoso-Moreira et al., 2020; Nolan et al., 2021; Zhao et al., 2024; Parvatam et al., 2025). The inability of animal models to fully replicate human disease is recognized as one of the key reasons why many drugs that appear effective in animals do not succeed in human clinical trials (Benam et al., 2015). These challenges highlight the need for novel approach methodologies (NAMs) that provide personalized and human-relevant model systems for studying disease mechanisms.

Over the past decade, advances in cell and molecular biology as well as in microtechnology have enabled the creation of complex human-cell-based *in vitro* and *in silico* models in two- or three-dimensional environments (2D, 3D), including patient-derived organoids and Organ-on-Chips. These models may replicate disease mechanisms at the cellular and tissue level, allowing researchers to evaluate basic biology, drug responses, and toxicity within the context of an individual patient’s genetic background (Baran et al., 2022; Parvatam et al., 2025; Weber et al., 2025). Although NAMs offer the possibility to model complex biology, the reproducibility of NAMs remains a concern. Utilization of animal-derived components with batch-to-batch variation and inter-species differences is one of the causes for low reproducibility (Oredsson et al., 2019). To ensure improved reproducibility and human relevance, the process of producing and analyzing NAMs should preferably be animal-free and with defined conditions (Weber et al., 2024). Furthermore, eliminating dependence on animal-derived materials is an ethical choice affecting the lives of millions of animals used in the production of, e.g., fetal bovine serum (FBS), antibodies, and bovine serum albumin (BSA) (Weber et al., 2022).

Serum acts as a common media supplement, increasing cell growth in an *in vitro* environment (Karnieli et al., 2017). The most widely used sera in cell culture medium include FBS (Arora, M., 2013), and human serum (HS) (Weber et al., 2022), providing essential nutrients, proteins, hormones, amino acids, and vitamins for the cells. FBS is recognized as an undefined, animal-derived supplement (Gstraunthaler, 2003) with batch-to-batch variation caused by the age and geographical differences in donor animals, in addition to biosafety risks such as endotoxins and mycoplasma contamination, besides serious ethical issues regarding animal welfare (Gstraunthaler et al., 2013). HS can offer an ethical advantage over FBS with more human-relevant culture conditions for *in vitro* disease modeling, but it consists of undefined components. Importantly, utilization of human blood products demands compliance with informed consent, tracking, and following confidentiality and privacy protocols.

Animal-derived polyclonal antibodies are widely used in biomedical research. The traditional production of antibodies requires immunization of individual animals, leading to batch-to-batch variability, uncertainty regarding the precise antibody binding site, and possible non-specific binding to non-target antigens (Peltomaa et al., 2022). Specificity issues can also arise in monoclonal antibody production via the hybridoma technique, as a single hybridoma may carry multiple antibody-producing genes (Laustsen et al., 2021). Additional limitations of the hybridoma technique include its lengthy production time (Laustsen et al., 2021), in contrast to the animal-free phage display method that can be completed within weeks (Peltomaa et al., 2022). A total of 477,632 animals were used for routine production, including blood products and antibodies, in the European Union (EU) in 2022. Out of these, 49,309 mice were going through a painful ascites method for the collection of monoclonal antibodies (EU, Commission staff working document 2022) (European Commission, 2024). The EU Reference Laboratory for Alternatives to Animal Testing (EURL ECVAM) assessed the scientific validityof antibodies and non-antibody affinity reagents produced via animal-free methodologies (Viegas et al., 2020; Zuang et al., 2024). They concluded that animal-free methods in antibody production are scientifically valid and gave a recommendation on the replacement of animal-derived antibodies in research and diagnostics (Viegas et al., 2020). In this regard, recombinant antibodies can improve research reproducibility and are considered as a more animal-friendly alternative, even though animal-derived material can be used in the production process (Nessar et al., 2025). Antibodies generated via phase display represent a fully animal-free alternative, as they are selected solely through an *in vitro* process without the need to use animal-derived material (Guliy et al., 2023). Also, chemically defined, serum-free supplements are widely regarded as the gold standard. Although the limitations of serum-containing media have been recognized for decades, the development of serum-free alternatives remains a challenge. Recent advancements are driving innovation in this field, underscoring its importance in addressing ethical concerns and the need for media optimization (Wijerathna-Yapa et al., 2025). In recent years, various protocols for using animal-free products in cell culture medium have been reported (Weber et al., 2024; Oredsson et al., 2025), but few studies have so far provided comprehensive experimental validation (Rafnsdóttir et al., 2023; Malakpour-Permlid et al., 2025; Mickols et al., 2025).

This study aims to encourage a transition from animal-derived to animal-free *in vitro* modeling process. By evaluating recombinant antibodies across different human cell types, we investigated the reliability of recombinant antibodies in cell culture and immunocytochemical staining processes. Additionally, the objective of this study is to evaluate the effectiveness of animal-free serum replacement (SR) as a substitute for HS in cell culture using human bone marrow stem/stromal cells (BMSCs) and human umbilical vein endothelial cells (HUVECs), in monoculture and in co-culture models. Commercial SR is a human cell line-derived supplement for the serum-free cell culture process. It contains proteins produced by continuously growing hepatocyte-derived cell lines in a concentrated and purified form. To employ an immunocytochemical staining process, we compared animal-derived and recombinant antibodies under two blocking conditions using either BSA or animal-free blocking (AFB) solution. We tested recombinant antibodies in immunocytochemical staining of human adipose stem/stromal cells (ASCs) and HUVECs against alpha-smooth muscle actin (αSMA) and cluster of differentiation 31 (CD31), respectively. In parallel, we evaluated the specificity of animal-derived and recombinant neuronal antibodies, β-tubulin III, and human neurofilament heavy chain (NF-H) in their non-target cells, ASCs and HUVECs, to assess the potential non-specific binding. This approach allows us to investigate both the reliability of recombinant antibodies and the influence of animal-free reagents in cell culture and in immunocytochemical staining. This study aims to contribute to the development of defined, reproducible, and ethical *in vitro* modeling for biomedical and tissue engineering applications.

## 2 Materials and methods

### 2.1 Cells

#### 2.1.1 Human umbilical vein endothelial cells

HUVECs were isolated from umbilical cords received from planned cesarean section at the Department of Obstetrics and Gynecology, Tampere University Hospital, with donor consent. The study was conducted in accordance with the Ethics Committee of the Pirkanmaa Hospital District, Tampere (R13019). The isolation of HUVECs was carried out according to the previously established protocol (Jaffe et al., 1973), further modified by us (Sarkanen et al., 2010). HUVECs were thawed and seeded in Endothelial Cell Growth Medium-2 (EGM-2, with supplements Bulletkit CC-3162, Lonza Clonetics™, and EBM-2 medium CC-3156, Switzerland). The 2% v/v HS (Lot no. 39055023HU, Ref no. S-HUO-EU-011, Serana, Germany) was used instead of FBS provided separately in the supplement bullet kit. HUVECs were used with passages 4-6.

#### 2.1.2 Bone marrow stem/stromal cells and human adipose stem/stromal cells

BMSCs were isolated from bone marrow samples received from an orthopedic surgery at Tampere University Hospital, Department of Orthopedics and Traumatology. Bone marrow samples were collected with written informed consent from donors and processed under the ethical approval of the Ethics Committee of the Expert Responsibility Area at Tampere University Hospital, Tampere, Finland (R15174). BMSCs were cultured in α-modified minimum essential medium (α-MEM, Gibco™, Thermo Scientific, product no. 22561054, United States of America) supplemented with 5% HS (Serana, Germany), 5 ng/mL basic fibroblast growth factor-2 (bFGF-2, product no.130-093-840, Miltenyi Biotec, United States of America), and 100 U/ml penicillin and 100 μg/mL streptomycin (Pen/Strep, product no. DE17-602E, Lonza). BMSCs were used with passages 3-7.

ASCs were extracted from subcutaneous abdominal tissue samples received from Tampere University Hospital, Department of Plastic Surgery, with written consent from the donors, and processed in accordance with the ethical approval granted by the Ethics Committee of the Expert Responsibility area of Tampere University Hospital (R15161). The cells were isolated according to the previously described method (Kyllönen et al., 2013). ASCs were cultured in α-MEM supplemented with 5% HS, 100 U/ml penicillin, and 100 μg/mL streptomycin, similar to BMSCs. ASCs were used with passages 3-6.

### 2.2 Cell culture

To compare animal-derived and recombinant antibodies and different blocking reagents, ASCs and HUVECs were seeded in a 48-well plate (Costar 48-well cluster plate, Corning® CellBIND®) with seeding densities of 4,000 cells/cm^2^ and 6,000 cells/cm^2^, respectively, in ASC medium (α-MEM supplemented with 5% HS, 100 U/ml penicillin, and 100 μg/mL streptomycin) and EGM-2. Medium was changed once before fixing the cells on day 3. Three replicate wells were used for each combination of antibody type and blocking condition.

To establish a serum- and animal-free cell culture medium, we used SR-reagent Xplace H2 (Animal origin-free, Lot. 108102boy, Seamless Bio (Germany), Scinora GmbH (Switzerland)), which consists of proteins produced from human hepatocyte-derived cell lines. SR includes transport proteins (transferrin, low amount serum albumin, transthyretin, apolipoprotein B-100), adhesion factors (fibronectin, vitronectin, various laminins such as 511, 411 and 211), protease inhibitors (alpha-1-antitrypsin, alpha-2-macroglobulin, plasminogen activator inhibitor) and growth factors with notable amount of vascular endothelial growth factor A (VEGF-A), insulin-like growth factor 2 (IGF-2), transforming growth factor β1 (TGFβ1) and low amount of other growth factors. SR was compared to HS used in culture media in the 2D angiogenesis model and in BMSC/HUVEC monocultures. For monocultures, BMSCs and HUVECs were seeded at cell densities of 4,000/cm^2^ and 6,000/cm^2^, respectively, and for the angiogenesis model, with densities of 20,000/cm^2^ and 4,000/cm^2^, respectively. Cells were seeded in a 48-well plate (Thermo Fisher Scientific, Denmark) in BMSC medium (BMSCs) or EGM-2 (HUVECs, angiogenesis model) and cultured for 6 days. One day after seeding (day 2), SR was added to the SR-medium groups at 1:2000. The 1% HS was used in addition to 1:2000 SR in the first medium change (day 2) to avoid stress on the cells and gradually adapt them to the new conditions. SR was used at 1:1000 for changing medium on day 4 and at 1:500 on day 6. Since the angiogenesis model started to detach from the wells on day 6, it was not exposed to the 1:500 dilution for the same duration as for other ratios. N = 3 for all different conditions.

### 2.3 Immunocytochemical staining

The immunocytochemical staining protocol was performed on 3 technical replicates for each cell type/combination and culture condition. After 6 days of culture, cells were washed with Dulbecco´s Phosphate Buffered Saline (DPBS++, product no. 14040-091, Gibco, United Kingdom) and fixed and permeabilized with 4% paraformaldehyde (product no. 15713-S, Electron Microscopy Sciences) and 0.1% Triton X-100 (product no. T8787-50ML, Sigma-Aldrich, St. Louis, MI, United States) in DPBS for 15 min at room temperature, followed by four washes with DPBS. Non-specific binding was blocked with blocking buffer [1% BSA; product no. A7906-100G, Sigma-Aldrich, St. Louis, MI, United States), in DPBS] for 1 h at 4 °C. We used primary antibodies against CD31 (M0823, mouse, 1:400, Agilent Technologies Singapore Pte Ltd., Singapore) to identify the presence of endothelial cells, and αSMA (ab124964, rabbit, 1:400, Abcam, United Kingdom) as a pericyte/stromal cell marker. Primary antibodies were diluted in 1% BSA in DPBS and applied overnight at 4 °C. The samples were then washed 4 times with DPBS. Secondary antibodies (A21202, Invitrogen, Thermo Fisher Scientific, United States of America) and Alexa Fluor 568 donkey anti-rabbit IgG (A10042, 1:400, Invitrogen, Thermo Fisher Scientific, United States of America) were diluted 1:400 in DPBS with 1% BSA and applied for 1h at 4 °C. The samples were washed once, stained with 0.5 μg/mL DAPI (product no. D9542-10MG) for 5 min at room temperature, followed by one wash with DPBS and two washes with milliQ water. Samples were stored in DPBS and imaged using a DMi8 widefield microscope (Leica). The images were processed using Fiji/ImageJ software (Schindelin et al., 2012) by modifying brightness and contrast.

#### 2.3.1 Recombinant antibodies in immunocytochemical staining

To develop an animal-free immunocytochemical staining protocol, we compared the performance and specificity of animal-derived and recombinant antibodies and tested the usability of a plant-derived AFB reagent (Animal-Free Blocker, product no. SP-5030-250, Vector Laboratories Inc.) as a replacement for BSA-based blocking solution and in antibody dilutions. This study focuses on commercial, hybridoma-based recombinant primary and secondary antibodies with animal-free batch production and compares them with antibodies produced by repeated immunization of laboratory animals. The staining protocol was as described above, except for the first washes, DPBS was used instead of PBS++. Original antibodies and their dilutions used for this part are listed in Table 1.

**TABLE 1:** All antibodies used in the study (Section 2.3.1) with detailed information.

Target protein/cell type	Abbreviation	Product number/manufacturer	Host	Dilution of primary antibody	Secondary antibody/manufacturer	Dilution of secondary antibody
Smooth muscle α-actin/ASC	αSMA	Ab7817/Abcam Inc	Mouse/monoclonal antibody	1:600	A21121/Goat anti-mouse IgG1/Polyclonal secondary antibody/Invitrogen	1:800
Smooth muscle α-actin/ASC	αSMA	Ab7817/Abcam Inc	Mouse/monoclonal antibody	1:200	A11029/Goat anti-mouse IgG/Polyclonal secondary antibody/Invitrogen	1:300
Endothelial cell adhesion molecule/HUVEC	CD31	M0823/Dako	Mouse/monoclonal antibody	1:400	A21121/Goat anti-mouse IgG1/Polyclonal secondary antibody/Invitrogen	1:800
Beta-tubulin III/ASC & HUVEC	β-tubulin III	A01627/Thermo Fisher scientific Inc	Rabbit/Polyclonal antibody	1:500	A21206/Donkey anti-rabbit IgG/Polyclonal secondary antibody/Thermo Fisher scientific Inc	1:400
Neurofilament heavy chain/ASC & HUVEC	NF-H	N5389/Sigma-Aldrich®	Mouse/Monoclonal antibody	1:1000	A21202/Donkey anti-mouse IgG/Polyclonal secondary antibody/Thermo Fisher scientific Inc	1:400
Smooth muscle α-actin/ASC	αSMA	ab124964/Abcam Inc	Rabbit/recombinant monoclonal antibody	1:500	A27034/Goat anti-rabbit IgG/Recombinant Superclonal/Invitrogen	1:400
Endothelial cell adhesion molecule/HUVEC	CD31	Ab76533/Abcam Inc	Rabbit/recombinant monoclonal antibody	1:250	A27034/Goat anti-rabbit IgG/Recombinant Superclonal/Invitrogen	1:400
Beta-tubulin III/ASC & HUVEC	β-tubulin III	Ab52623/Abcam Inc.	Rabbit/recombinant monoclonal antibody	1:1000	A27034/Goat anti-rabbit IgG/Recombinant Superclonal/Invitrogen	1:100
Neurofilament heavy chain/ASC & HUVEC	NF-H	Ab207176/Abcam Inc.	Rabbit/recombinant monoclonal antibody	1:50	A27034/Goat anti-rabbit IgG/Recombinant Superclonal/Invitrogen	1:100

ASCs and HUVECs were immunocytochemically stained using both animal-derived and recombinant antibodies, as well as BSA-based and AFB and antibody dilution solution AFB that was used as a 1:4 dilution in DPBS. The performance of the recombinant antibodies was tested using antibodies against cell-type-specific markers: αSMA antibodies for ASCs, and CD31 antibodies for HUVECs. The specificity of the markers was assessed by staining HUVECs and ASCs with neuronal antibodies β-tubulin III and NF-H. Animal-derived primary antibodies were combined with animal-derived secondary antibodies, and recombinant antibodiesprimaries with recombinant antibodiessecondaries to employ a constant protocol. Images were acquired by using a wide-field fluorescence microscope (Olympus IX 51).

### 2.4 Statistical analysis

The fluorescence images were analyzed using Fiji/ImageJ software for nuclei counting and AngioTool 64 – Version 0.6a for vascular quantification. After uploading the images, the software automatically performs vessel segmentation. To prevent exaggerated or unrealistic segmentation, the adjustable parameters (e.g., diameter and intensity) were manually tuned on an image-by-image basis to reflect the original image data. AngioTool is a free, open-source software validated to compute several vascular parameters (Zudaire et al., 2011). To ensure reproducibility, three biological images per condition were analyzed. All data are represented as mean ± SD. Statistical analysis was performed using GraphPad Prism 9.0.0. (GraphPad Software, www.graphpad.com). Unpaired Student’s T-tests were used for significance testing for BMSCs, HUVECs, and BMSCs + HUVECs in HS- and SR-supplemented media. The differences were considered statistically significant for p < 0.05 and represented as follows: * denotes p < 0.05, **p < 0.01, ***p < 0.001, and ‘ns’ as a non-significant.

## 3 Results

### 3.1 Serum replacement supplemented medium supported the growth of BMSCs but not HUVECs

**FIGURE 1:**
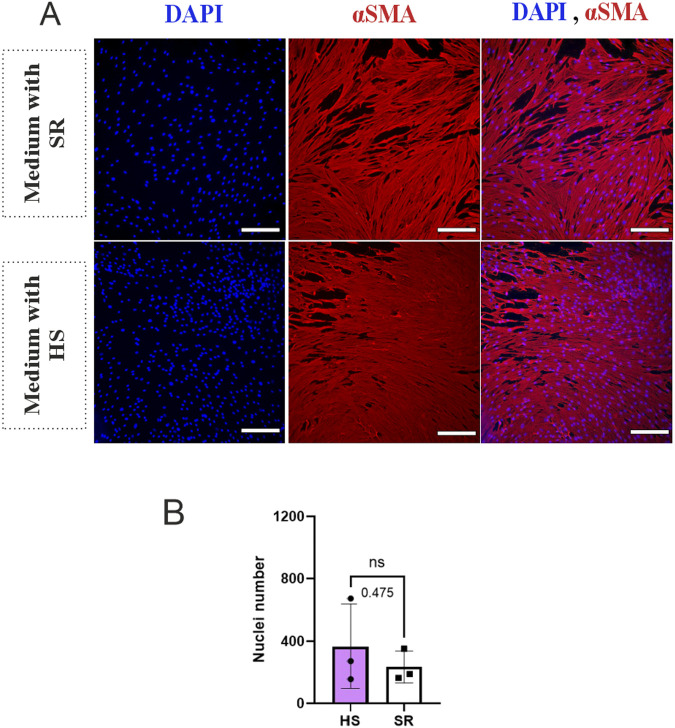
The number of nuclei and morphology of Bone marrow stem/stromal cells (BMSCs) in media supplemented with human serum (HS) and serum replacement (SR) at day 6, assessed with immunocytochemical staining. **(A)** Morphology of BMSCs, Blue color: Nuclei, Red color: alpha-smooth muscle actin (αSMA). **(B)** Number of nuclei in the BMSCs monoculture. Statistical analysis was performed using a t-test. Data are presented as mean ± SD. ‘Ns’ denotes a non-significant difference. Magnification: ×10, Scale bar: 100 μm, N = 3.

**FIGURE 2:**
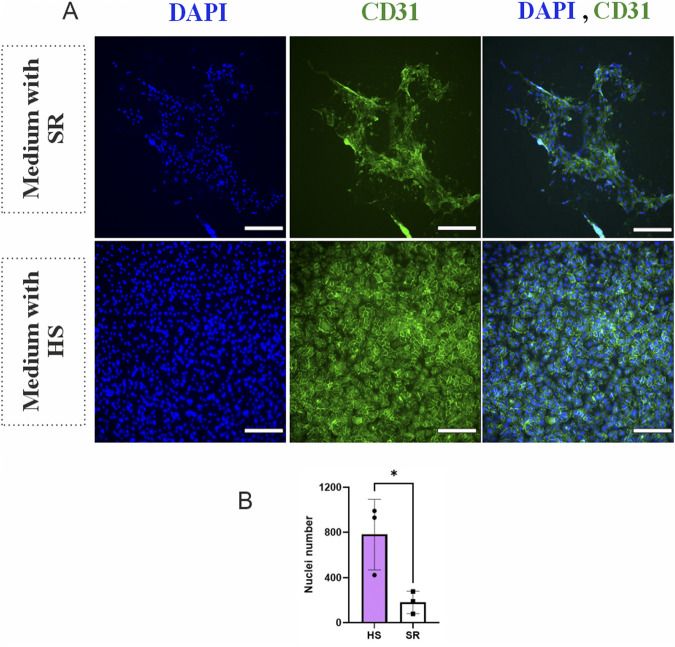
The number of nuclei and morphology of Human umbilical vein endothelial cells (HUVECs) in media supplemented with human serum (HS) and serum replacement (SR) at day 6, assessed with immunocytochemical staining. **(A)** Morphology of HUVECs, Blue color: Nuclei, Green color: cluster of differentiation 31(CD31). **(B)** Number of nuclei in the HUVECs monoculture. Statistical analysis was performed using a t-test. Data are presented as mean ± SD. Statistical significance denoted with a single asterisk (^*^) for p ≤ 0.05. Magnification: ×10, Scale bar: 100 μm, N = 3.

To evaluate cell growth, morphology, and vascular network formation capacity, BMSCs and HUVECs were cultured in their cell-specific medium with HS or with SR. Figure 1A shows the morphology of BMSCs in media containing HS versus SR on day 6 after immunocytochemical staining. During culture, BMSCs demonstrated an elongated, spindle-like morphology in both media. In the medium containing SR, cells appeared less compact than those in HS, although these differences were minor when visually inspected. During the 6-day culture period, cell expansion and morphology remained constant in both media. Figure 1B shows the number of BMSCs as counts of nuclei, indicating no significant difference between the number of cells in different media. Figure 2A illustrates the morphology of HUVECs and shows highly variable behavior of the cells in the presence of HS and SR. HUVECs cultured in medium containing HS demonstrated higher cell density and more uniform distribution of CD31 endothelial marker, indicating improved proliferation, while HUVECs cultured in medium containing SR showed a notably lower cell density and less uniformity in cell distribution. Figure 2B confirms the significantly higher number of HUVECs in the presence of HS compared to the SR-supplemented medium (p ≤ 0.05).

### 3.2 Vascular network formed in the presence of serum replacement

**FIGURE 3:**
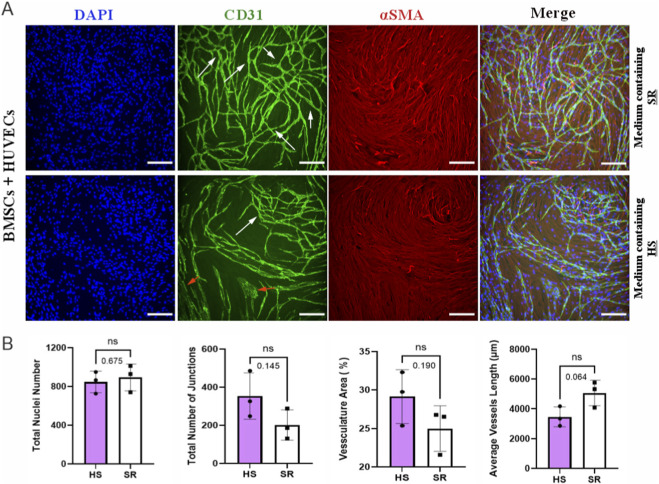
Comparing vascular network formation and morphology. **(A)** Morphology of co-culture of bone marrow stem/stromal cells with human umbilical vein endothelial cells (BMSCs + HUVECs) in media supplemented with human serum (HS) and serum replacement (SR) at day 6, assessed with immunocytochemical staining. Orange arrows indicate the unorganized endothelium, and white arrows show vascular network formation. Blue color: Nuclei, Green color: cluster of differentiation 31(CD31), Red color: alpha-smooth muscle actin (αSMA). **(B)** quantification of the total number of nuclei of BMSCs + HUVECs co-culture, the total number of junctions, the vasculature percentage area (%), and the average vessel length (µm) of the green channel in BMSCs + HUVECs co-culture. Statistical analysis was performed using a t-test. Data are presented as mean ± SD. ‘Ns’ denotes a non-significant difference. Magnification: ×10, Scale bar: 100 μm, N = 3.

Vascular parameters were visually evaluated as indicators of cell behavior and vascular network formation capacity in a medium containing HS or SR in a co-culture model. Figure 3A shows the results of BMSCs + HUVECs, indicating the vascular network capacity of the co-culture model. The results demonstrated that BMSCs + HUVECs co-culture in the presence of HS formed vascular network structures, but also unorganized endothelium, as shown with orange arrows in Figure 3A. Interaction with vascular supporting cells (BMSCs) was visible in the vascular network, as shown by white arrows. The vascular network formed in the presence of SR was mainly similar in structure (white arrows) but lacked the unorganized endothelium. Branching and uniform distribution of vascular network throughout the cell culture area was visible in both media, as shown with nuclei, anti-smooth muscle actin (αSMA, red), and anti-CD31 (green) staining. Figure 3B compares the total number of nuclei, the vasculature area, the total number of vascular junctions, and the average vascular length formed by BMSCs + HUVECs in both media. Statistical analysis shows no significant difference between the vascular parameters in the presence of HS and SR.

### 3.3 Performance and specificity of recombinant antibodies in the immunocytochemical staining of cells

**FIGURE 4:**
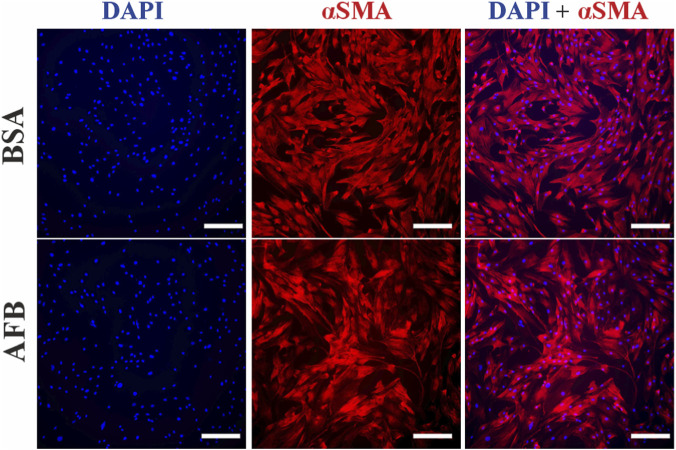
Fluorescence images of immunocytochemical staining process of Human adipose stem/stromal cells (ASCs) stained with recombinant antibodies treated with unspecific binding blocked with either bovine serum albumin (BSA) or animal-free blocker (AFB). Blue color: Nuclei, Red color: alpha-smooth muscle actin (αSMA). Magnification: ×10, Scale bar: 100 µm.

**FIGURE 5:**
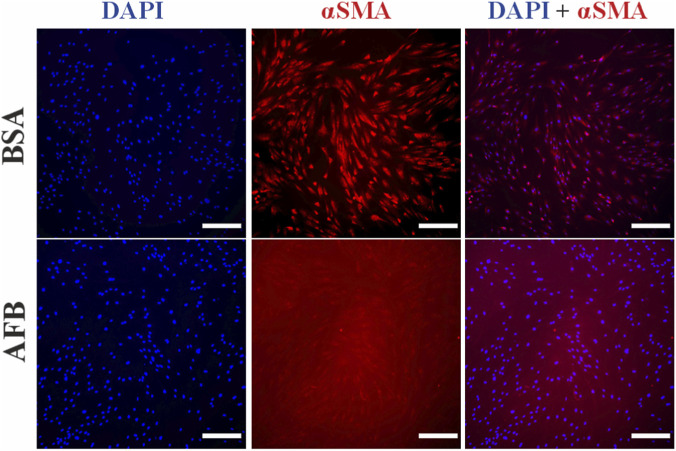
Fluorescence images of immunocytochemical staining process of Human adipose stem/stromal cells (ASCs) stained with animal-derived antibodies treated with unspecific binding blocked with either bovine serum albumin (BSA) or animal-free blocker (AFB). Blue color: Nuclei, Red color: alpha-smooth muscle actin (αSMA). Magnification: ×10, Scale bar: 100 µm.

We assessed the immunocytochemical staining process with unspecific antibody binding blocked with either BSA or AFB, followed by staining with either recombinant antibodies or animal-derived antibodies. In ASCs stained with recombinant antibodies against αSMA, fluorescence signal from the cytoskeleton was observed under both BSA and AFB blocking conditions. Targeted binding of the antibody was confirmed with cell-specific morphology with an elongated, spindle-shaped structure (Figure 4). Similarly, when ASCs were stained with an animal-derived antibody against αSMA, a cytoskeletal signal was detected with both blocking solutions. However, images obtained with AFB blocking solution showed higher background fluorescence and reduced clarity compared to BSA blocking (Figure 5). DAPI staining confirmed that the cell number was comparable across all stained samples.

**FIGURE 6:**
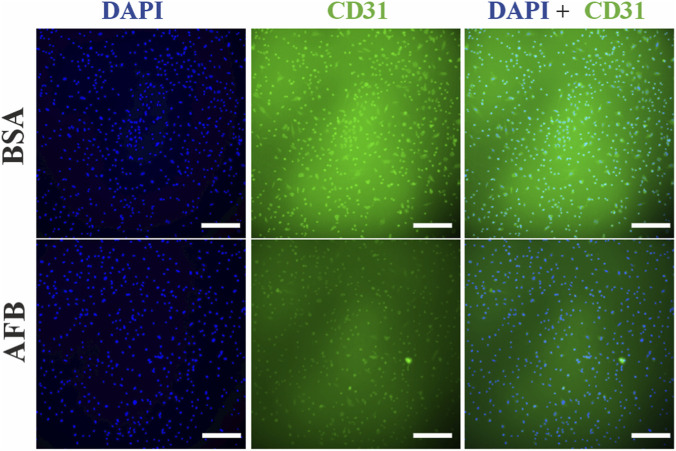
Fluorescence images of immunocytochemical staining process of Human umbilical vein endothelial cells (HUVECs) stained with recombinant antibodies treated with unspecific binding blocked with either bovine serum albumin (BSA) or animal-free blocker (AFB). Blue color: Nuclei, Green color: cluster of differentiation 31(CD31). Magnification: ×10, Scale bar: 100 µm.

**FIGURE 7:**
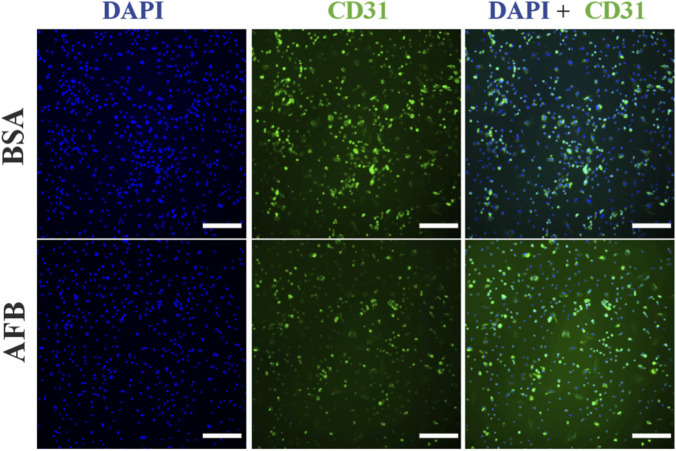
Fluorescence images of immunocytochemical staining process of Human umbilical vein endothelial cells (HUVECs) stained with animal-derived antibodies treated with unspecific binding blocked with either bovine serum albumin (BSA) or animal-free blocker (AFB). Blue color: Nuclei, Green color: cluster of differentiation 31(CD31). Magnification: ×10, Scale bar: 100 µm.

In HUVECs stained with recombinant antibodies against CD31 located in the endothelial cell surface, a fluorescence signal was detected under both blocking conditions. However, incomplete staining of the cell surface was observed with signal overlapping with nuclei staining (Figure 6). Similarly, in HUVECs stained with animal-derived antibody against CD31, fluorescence signal was observed in both BSA- and AFB-blocked solutions close to the cell nuclei instead of the cell surface (Figure 7). DAPI staining confirmed that the cell number was comparable across all samples.

**FIGURE 8:**
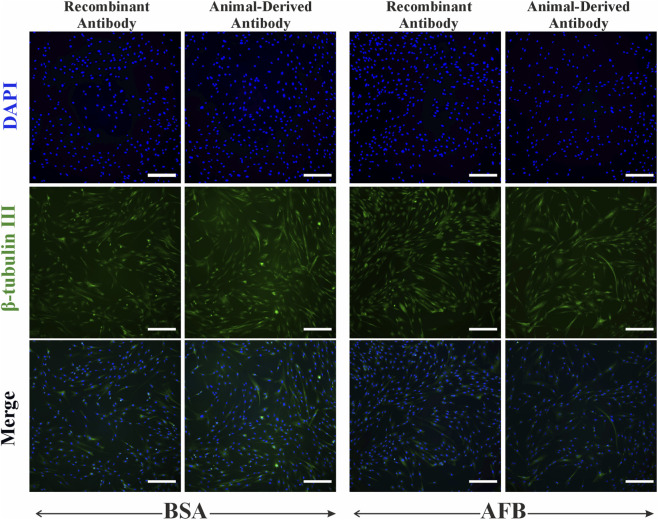
Fluorescent imaging of Human adipose-derived stem/stromal cells (ASCs), stained with β-tubulin III neural antibody, either recombinant antibody or animal-derived antibody types, and blocked with two solutions: bovine serum albumin (BSA) and animal-free (AFB). Blue color: Nuclei, Green color: β-tubulin III. Magnification: ×10, Scale bar: 100 µm.

**FIGURE 9:**
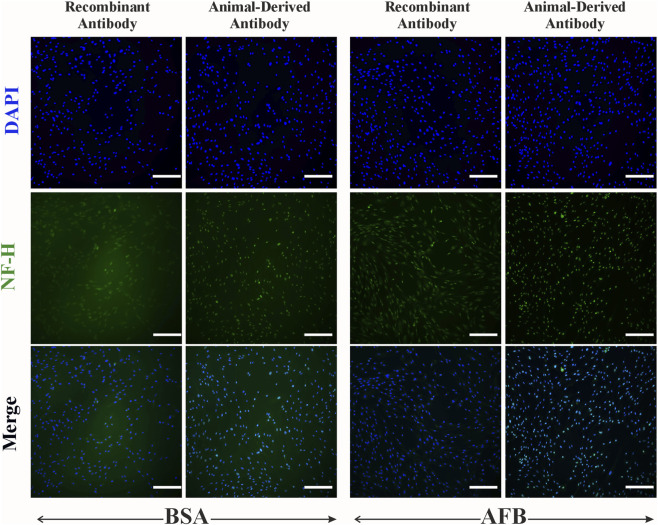
Fluorescent imaging of Human adipose-derived stem/stromal cells (ASCs), stained with human neurofilament heavy chain (NF-H) antibody, either recombinant antibody or animal-derived antibody types, and blocked with two solutions: bovine serum albumin (BSA) and animal-free (AFB). Blue color: Nuclei, Green color: NF-H. Magnification: ×10, Scale bar: 100 µm.

**FIGURE 10:**
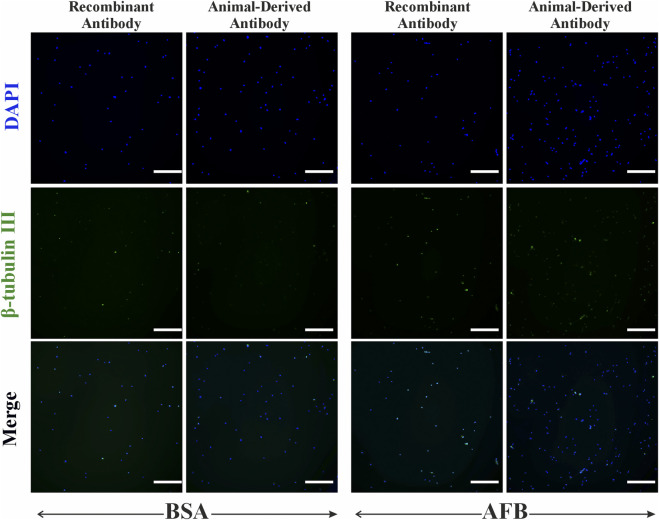
Fluorescent imaging of Human umbilical vein endothelial cells (HUVECs), stained with β-tubulin III neural antibody, either recombinant antibody or animal-derived antibody types, and blocked with two solutions: bovine serum albumin (BSA) and animal-free (AFB). Blue color: Nuclei, Green color: β-tubulin III. Magnification: ×10, Scale bar: 100 µm.

**FIGURE 11:**
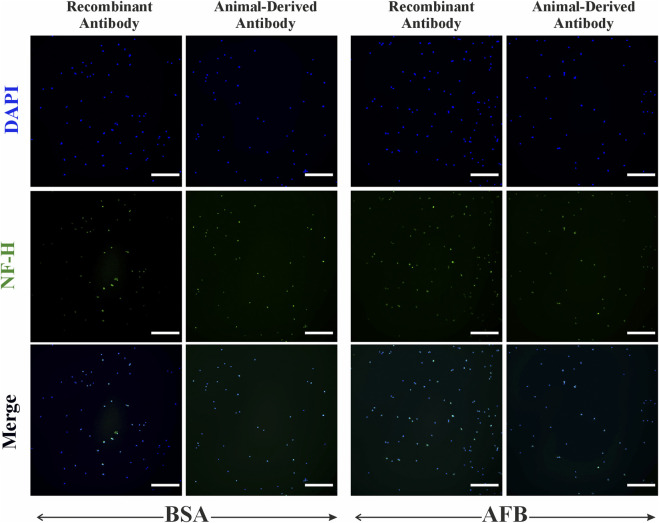
Fluorescent imaging of Human umbilical vein endothelial cells (HUVECs), stained with human neurofilament heavy chain (NF-H) antibody, either recombinant antibody or animal-derived antibody types, and blocked with two solutions: bovine serum albumin (BSA) and animal-free (AFB). Blue color: Nuclei, Green color: NF-H. Magnification: ×10, Scale bar: 100 µm.

Immunocytochemical staining of ASCs against neural antibodies β-tubulin III and NF-H was performed to assess non-specific binding of these antibodies in non-target cells. For β-tubulin III (Figure 8), we detected partial non-specific binding of recombinant antibodies and animal-derived antibodies in ASCs. NF-H staining in ASCs (Figure 9) showed some fluorescence signaling overlapping with nuclei and minor non-specificity independent on the antibody source and blocking solution (Figure 9). Besides some fluorescence signals overlapping with nuclei, HUVECs showed no detectable non-specific binding of β-tubulin III (Figure 10) or NF-H antibodies (Figure 11) regardless of origin and blocking solution used.

## 4 Discussion

This study assessed several animal-free components, including SR, recombinant antibodies, and AFB solution, used in cell culture and immunocytochemical staining of cells to increase reproducibility and to avoid ethical problems related to animal-derived products. Here, we provide insights on how researchers can do a controlled shift from animal-derived cell culture process towards animal-free *in vitro* modeling while increasing the specificity of their antibodies (Baker, 2015; Bradbury and Plückthun, 2015).

The growth, morphology, and vascular network formation capacity of BMSCs and HUVECs were evaluated in the presence of HS and SR in monoculture and in co-culture models with the aim to assess the performance of SR and to provide more defined and *vivo*-like culture conditions. Commercial SR reagent includes transport proteins (Transferrin, serum albumin in low amount, transthyretin, apolipoprotein B-100), adhesion molecules (fibronectin, vitronectin, and various laminins such as 511, 411, and 211), and a low concentration of growth factors, but a notable amount VEGF-A, IGF-2, and TGFβ1, and was used as an alternative to HS in an angiogenic 2D model. Despite the fact that medium supplemented with serum supports the growth and survival of several different cell types, it can lead to decreased reproducibility of research results and an increased risk of microbial contamination (Yao and Asayama, 2017). Our results showed distinct morphological and proliferative differences between SR- and HS-supplemented cell cultures. Although SR can support BMSC growth with a typical spindle-shaped morphology, HS promoted cell growth in a slightly improved manner, likely because of its complex combination of growth factors and adhesion molecules. According to a previous report, HS has a significant amount of growth factors and bioactive compounds, including platelet-derived growth factor (PDGF) (Mizuno et al., 2015), enabling the growth of mesenchymal stem/stromal cells (MSCs) without altering their characteristics or functional properties (Thaweesapphithak et al., 2019). A previous study compared adipose-derived mesenchymal stem cells cultured in FBS-containing and serum-free media and reported that serum-free culture offers advantages in culture time, genetic stability, multipotentiality, and immunogenicity (Lee et al., 2022). Compared to BMSCs, HUVECs showed more prominent differences. Under HS conditions, endothelial cells proliferated efficiently without visible de-differentiation, whereas in SR conditions, the growth and survival of cells were poor, possibly because of stress reactions due to reduced medium. Although SR contains essential growth factors, we did not observe a supportive effect on HUVECs’ growth and proliferation. Our observation is in accordance with previous reports highlighting the need for a balance between growth factors and supplements to support the growth of HUVEC (Marin et al., 2001; Baudin et al., 2007; Duranova et al., 2024). As an example, SR contains TGFβ1, which is considered a regulator of tissue development and suppression of proliferation in many cell types (Massagué and Sheppard, 2023). Although TGFβ1 supports angiogenesis *in vivo*, it can induce endothelial cell apoptosis (Roberts et al., 1986; Pollman et al., 1999; Ferrari et al., 2009). Because of the TGFβ1’s inhibitory impact on the endothelial cells, it is reported that TGFβ1 indirectly supports the angiogenesis process *in vivo* by inducing the VEGF expression or other factors involved in angiogenesis (Pardali and Moustakas, 2007; Ferrari et al., 2009). VEGF-A is a well-known angiogenesis inducer (Hicklin and Ellis, 2005), but needs a balanced concentration with other growth factors present in SR.

The lack of essential mitogenic and survival factors is likely one reason behind the limited proliferation and survival of HUVECs in the monoculture model supplemented with SR. HS contains bFGF, epidermal growth factor (EGF), insulin-like growth factor-1 (IGF-1), vascular endothelial-derived growth factors, and PDGF (Koellensperger et al., 2006) which have been shown to enhance the expansion and viability of HUVECs *in vitro* (Dunn et al., 2000; Nourse et al., 2007). Also, the hepatocyte-derived nature of the SR may result in a metabolic and signaling profile that does not fully support the specific needs of primary endothelial cells, and its formulations may lack critical, niche-specific components found in HS. Interestingly, while HUVECs could not grow and proliferate optimally in monoculture in medium supplemented with SR, they formed vascular structures when co-cultured with BMSCs in the presence of SR. This finding highlights the importance of paracrine signals, including VEGF, bFGF, and matrix remodeling enzymes secreted by mesenchymal stem/stromal cells, which together facilitated endothelial survival, migration, and vascular organization (Kocherova et al., 2019; Chen et al., 2024). This observation highlights a pivotal point in the transition toward an animal- and serum-free culture system, as the limitations observed in monoculture should not be considered a definitive barrier to implementing animal- and serum-free reagents. Instead, our findings suggest that more complex, physiologically relevant models, such as BMSCs + HUVECs co-culture, can provide the necessary support to overcome the challenges of serum-free environment. We hypothesize that cellular monocultures require more optimization and extensive growth factor-based support, typically received from serum, whereas co-cultures can be treated with reduced medium due to paracrine effects and the support of other cell types. When visually inspected, we could detect more vascular branching in the presence of SR with a more defined network structure compared to the HS-supplemented medium. However, the statistical evaluation of nuclei number and vascular parameters reveals no significant differences between the media, highlighting the successful transition to using serum-free cell culture medium in a 2D environment.

The EU promotes the Replacement, Reduction, and Refinement (the 3R´s) of animals used for scientific purposes. Traditional antibody production by repeated immunization of laboratory animals, typically mice, requires painful collection of target proteins from ascites. Animal-free methods, based on e.g., phage display technique, can produce antibodies without the need to use animals. New methods avoid ethical issues and can also improve antibody specificity by increasing antigen binding accuracy (Gray et al., 2020). Completely animal-free antibodies require production based on a known antibody gene sequence, followed by *in vitro* expansion of the target protein in non-animal host organisms within a process that contains no animal-derived components. While powerful, these methods are time-consuming and technically complex. Currently, there are only a few completely animal-free monoclonal or polyclonal antibodies available on the market. Several recombinant antibodies are available, and these are typically produced in hybridomas. Hybridomas are a fusion of antibody-producing B cells with immortal myeloma cells, thus creating cell lines that can offer a continuous supply for specific antibodies (Mak and Saunders, 2006). However, hybridomas typically rely on animal-derived B cells in their establishment and, even though producing animal-free batches, is not considered completely animal-free technology. Also, genetic drift remains a concern since antibody specificity can be lost during long-term hybridoma culture.

EURL ECVAM does not recommend discontinuing the use of existing well-characterized hybridomas, but encourages sequencing efforts and use of characterized hybridomas in recombinant antibody production (Gray et al., 2020). Establishing new hybridomas that produce antibodies already available or in the market should be avoided due to the need to use of animals in the early process. Continued education, improved accessibility, and institutional support are essential to enable a gradual transition toward fully animal-free antibodies without constraining scientific research (Gray et al., 2020).

When comparing animal-derived and recombinant antibodiesin the immunocytochemical staining process, we employed both animal-derived BSA and animal-free AFB solutions to block the unspecific binding of antibodies. Furthermore, the same blocking solution was used in antibody dilutions. Our findings showed distinct differences in antibody performance and fluorescence signal pattern between the different protocols and blocking solutions. In ASC, the recombinant antibodiesantibody against αSMA provided a stronger fluorescence signal compared to the animal-derived equivalent under both blocking conditions. The strongest fluorescence signal was achieved when the recombinant antibodieswas used with BSA, with a comparable cell amount confirmed with nuclei staining. Our results indicate that the antibody production method can substantially influence binding efficiency and detection sensitivity shown in the differences in fluorescence signals. A previous study demonstrating variability in immunoreactivity between recombinant and traditional animal-derived antibodies has shown that recombinant antibodies showed comparable biological activity to their ascites-derived parent antibodies and could serve as an alternative to animal-derived monoclonal antibodies (Wang et al., 2024). CD31-stained endothelial cells demonstrated unspecific binding as an overlapping signal with the nuclei and absence of CD31 antigen located on the cell surface in almost all stained samples. This could indicate suboptimal antigen preservation or accessibility under the fixation or permeabilization protocol used, or a low expression of CD31 in the tested culture condition. The fact that both αSMA and CD31 detection were influenced by the combination of antibody source and blocking agent underscores the importance of optimizing immunostaining protocols for animal-free reagents.

Finally, we wanted to perform an independent experiment to assess the possible unspecific binding of neuronal antibodies β-tubulin III and NF-H to their non-target cells, i.e. ASCs and HUVECs. In ASCs, β-tubulin III antibodies, whether animal-derived or recombinant antibodies, demonstrated partial non-specific binding to intracellular structures, likely associated with cytoskeletal components. NF-H antibodies exhibited minor unspecificity independently of the antibody origin. A previous study noted that BSA can prevent non-specific binding by blocking hydrophobic interactions and electrostatic forces between proteins and tissue components (Buchwalow et al., 2011; Modi, 2025). In HUVECs, we did not detect non-specific binding of β-tubulin III or NF-H besides some overlapping signals with nuclei. Taken together, these results highlight the necessity of optimizing antibody origin and blocking conditions to maintain antibody specificity.

## 5 Conclusion

The use of animals in experimental research, whether as model organisms or as a source of reagents, is ethically problematic and often fails to yield reproducible and reliable results relevant to human physiology and diseases. This study explored the potential of animal-free alternatives, including SR, AFB reagents, and recombinant antibodies, to improve scientific accuracy and ethical responsibility. Our evaluation of antibody specificity revealed that antibody origin, blocking strategy, and cell type critically influence the outcome. The results suggest that SR may be suitable for certain cell types, but this needs to be confirmed in a cell-type-specific manner. Our findings emphasize that while the adoption of serum- and animal-free reagents is a crucial step toward ethical and sustainable research, careful optimization is essential to maintain experimental accuracy.

## Data Availability

The raw data supporting the conclusions of this article will be made available by the authors, without undue reservation.
